# Plasma levels of the arterial wall protein fibulin-1 are associated with carotid-femoral pulse wave velocity: a cross-sectional study

**DOI:** 10.1186/1475-2840-12-107

**Published:** 2013-07-18

**Authors:** Esben Laugesen, Pernille Høyem, Jens Sandahl Christiansen, Søren Tang Knudsen, Klavs Würgler Hansen, W Scott Argraves, Troels Krarup Hansen, Per Løgstrup Poulsen, Lars Melholt Rasmussen

**Affiliations:** 1 Department of Endocrinology and Internal Medicine, Aarhus University Hospital, Noerrebrogade 44, 8000 Aarhus C, Denmark; 2 Medical Department, Diagnostic Center, Regional Hospital Silkeborg, Falkevej 1-3, 8600 Silkeborg, Denmark; 3 Department of Regenerative Medicine and Cell Biology, Medical University of South Carolina, 173 Ashley Avenue, Charleston, SC 29425, USA; 4 Department of Clinical Biochemistry and Pharmacology, Institute of Molecular Medicine and Clinical Institute, University of Southern Denmark, Sdr. Boulevard 29, 5000 Odense, Denmark

**Keywords:** Type 2 diabetes mellitus, Arterial stiffness, Pulse wave velocity, Fibulin-1

## Abstract

**Background:**

The arterial system in diabetic patients is characterized by generalized non-atherosclerotic alterations in the vascular extracellular matrix causing increased arterial stiffness compared with subjects without diabetes. The underlying pathophysiology remains elusive. The elastin-associated extracellular matrix protein, fibulin-1, was recently found in higher concentrations in the arterial wall and in plasma in patients with long duration type 2 diabetes. Furthermore, plasma fibulin-1 independently predicted total mortality and was associated with pulse pressure, an indirect measure of arterial stiffness. Whether plasma fibulin-1 is associated with arterial stiffness at earlier phases of type 2 diabetes has not been determined.

**Methods:**

In this cross-sectional study, we examined 90 patients with recently diagnosed type 2 diabetes (< 5 years) and 90 gender- and age-matched controls. Plasma fibulin-1 was measured immunochemically. Arterial stiffness was assessed by carotid-femoral Pulse Wave Velocity (PWV). Differences in means were assessed by t-tests. Associations were assessed by multivariate regression analyses.

**Results:**

Plasma fibulin-1 levels were lower in the diabetic group compared with the control group, 93 ± 28 vs 106 ± 30 μg/mL, p = 0.005. In unadjusted analysis of the total study sample, plasma fibulin-1 was not associated with PWV, p = 0.46. However, with adjustment for the confounders age, gender, mean blood pressure, heart rate, body mass index, diabetes and glomerular filtration rate, a 10 μg/mL increase in plasma fibulin was associated with 0.09 ± 0.04 m/s increase in PWV, p < 0.05. In subgroup analysis, plasma fibulin-1 was associated with PWV in the diabetes group, (0.16 ± 0.07 m/s increase in PWV per 10 μg/mL increase in plasma fibulin-1, p<0.05), but not controls, β = 0.021 ± 0.057 m/s per 10 μg/mL, p = 0.70. The association remained significant in the diabetes group after adjustment for covariates, p < 0.05.

**Conclusions:**

Plasma fibulin-1 is independently associated with PWV. Yet, as the plasma level of fibulin-1 was lower in patients with recently diagnosed type 2 diabetes than in healthy controls, plasma fibulin-1 levels are not a simple marker of the degree of arterial stiffening. Further studies are needed to determine the exact role of fibulin-1 in arterial stiffness and cardiovascular risk in patients with type 2 diabetes.

## Introduction

Despite the intensified blood pressure and cholesterol treatment during the previous decades, type 2 diabetes (T2DM) remains associated with an increased risk of fatal and non-fatal cardiovascular disease (CVD) [1,2]. The pathophysiological processes underlying this association remains elusive. Arterial wall stiffness may be a contributing factor. Arterial stiffness has been shown to predict cardiovascular disease [3] and the vascular system in patients with type 2 diabetes is characterized by an increased stiffness of the arterial wall compared to subjects without diabetes [4]. The pathophysiology underlying the increased arterial stiffening seen in diabetes patients probably involves changes in structure and amount of extracellular matrix (ECM) molecules in the media layers of the arteries, including collagens and elastin [5-10], but remains overall incompletely understood. Interestingly, the elastin-associated ECM molecule fibulin-1 was recently identified in non-atherosclerotic arterial tissue from patients with type 2 diabetes [11]. The fibulin-1 protein was found in higher concentration in the arterial wall and in plasma from patients with type 2 diabetes with long diabetes duration and CVD, compared with non-diabetics with CVD [11]. Moreover, in T2DM patients with longstanding disease, fibulin-1 was associated with pulse pressure, an indirect measure of arterial stiffness, and independently predicted total mortality during 15 years of follow-up [11]. Fibulin-1 levels in recently diagnosed T2DM patients have not yet been described.

Carotid-femoral pulse wave velocity (PWV) is considered the gold standard for non-invasive assessment of arterial stiffness [3]. Increased PWV is independently associated with cardiovascular morbidity and mortality in both low- and high-risk non-diabetic populations [12-18] and predicts cardiovascular and total mortality in patients with type 2 diabetes [19]. The association between fibulin-1 and PWV in patients with type 2 diabetes remains to be elucidated.

Thus, in this study we sought to i) compare the plasma-fibulin-1 levels in patients with recently diagnosed type 2 diabetes with a gender- and age-matched control group and ii) to study the association between plasma fibulin-1 levels and PWV.

### Subjects and methods

100 patients with type 2 diabetes and 100 controls matched individually for gender and age were included in the present study. The study protocol has been described in detail elsewhere (Clinical Trials no NCT00674271). Briefly, the patients were consecutively recruited from the outpatient clinic at Aarhus University Hospital, Denmark. Inclusion criteria were age >18 years, diagnosis of type 2 diabetes according to WHO criteria [20] and known duration of diabetes < 5 years. The control subjects were recruited by advertising in the local press and matched consecutively by gender and age to the diabetes patients. Undiagnosed diabetes was excluded by fasting glucose and oral glucose tolerance test. Subjects with impaired fasting glucose (9 participants) or impaired glucose tolerance (3 participants) or both (2 participants) were accepted as control subjects. Exclusion criteria for both patients with diabetes and controls were acute or chronic infectious disease, end-stage renal failure, pregnancy or lactation, prior or present cancer and contraindications to magnetic resonance imaging (MRI) (claustrophobia, magnetic material in the body and body weight >120 kg). History of hypertension or cardiovascular disease were not exclusion criterions. PWV data were not recordable in 4 patients owing to atrial fibrillation, in 3 patients owing to obesity and in 2 patients owing to technical problems. Fibulin-1 data was not available in one patient due to technical problems (only a minor quantity of blood could be drawn from the patient, insufficient for analysis beyond baseline data). The 10 matches for these 10 participants were also excluded; thus, data from 180 participants were available for statistical analysis. The study was approved by the Research Ethics Committee of Central Region, Denmark and by the Danish Data Protection Agency. All patients gave their written informed consent to participate.

### Biochemical analysis

Fibulin-1 was analyzed with a sandwich immunoassay [11] modified from a previously described method [21]. Briefly, 96-wells were coated with 3 μg/ml rabbit anti human fibulin-1 IgG (Rb 2954 IgG) and blocked with 3 mg/ml BSA in TBS. Plasma samples, diluted 1/1.000, were added to wells for 2 hours, washed and anti-human fibulin-1 IgG (mouse monoclonal, 3A11) applied and incubated for 2 hours at room temperature. Detection of bound secondary antibody was done with rabbit anti mouse-IgG (AD0124, PerkinElmer), labeled with Europium (Eu). Bound Eu was detected using time-resolved fluorescence on DELFIA (Perkin Elmer). A standard curve (ranging from 0.01-0.32 μg protein/ml) was generated using purified human fibulin-1. The linearity of the immunoassay was tested using two plasma samples, diluted from 1/200 to 1/2,000. Interassay variation was ~10%.

### Pulse wave velocity measurements

Examinations were conducted between 9 a.m. and 1 p.m. after a minimum of 5 minutes rest in a quiet room. The study subjects had abstained from smoking and intake of tea, coffee or other caffeine-containing beverages for at least 3 hours prior to the examinations. At least two hours elapsed between breakfast and the examinations.

Measurements of PWV were performed using an applanation tonometer (Millar, SPT-301B, Houston, Texas, USA) and SphygmoCor equipment and software, version 8.0 (AtCor Medical, Sydney, Australia). After a minimum of 5 minutes of rest in the supine position, the carotid-femoral PWV was determined by sequential ECG referenced recordings of the pulse wave at the carotid and the femoral artery by the tonometer. The transit time was determined by the intersecting tangent algorithm method [22] and the path length calculated by subtracting the distance between the carotid artery measurement site and sternal notch from the distance between the femoral artery site and the sternal notch [3], all measured directly by a tape measure. The mean of two PWV measurements was calculated for each patient. Within-subject coefficient of variation for PWV was 5.1%.

Office blood pressure (BP) was measured on the right arm, and mean systolic and diastolic BP were calculated as the average of 3 measurements obtained after a minimum of 5 minutes of rest in the seated position. BP was measured by a Riester Champion N automatic blood pressure monitor (Riester GmbH, Jungingen, Germany) or a Speidel&Keller mercury sphygmomanometer (Speidel&Keller, Welch Allyn, Jungingen, Germany). Pulse pressure (PP) was calculated as systolic BP minus diastolic BP, and mean arterial pressure (MAP) as diastolic BP + ((systolic minus diastolic BP)/3).

24-h Ambulatory BP monitoring (ABPM) was performed using Spacelabs 90217 (Spacelabs Healthcare, Issaquah, Washington, USA). BP was measured with 20 minute intervals day and night. The calculation of day and night BP was based on patients’ individual diary recordings of awake and sleeping hours. Recordings with more than three missing hours (maximum 1 hour during nighttime) were excluded from the analysis.

### Statistical analyses

Differences in means between the diabetes and control group were assessed by paired t-tests. Differences within groups were assessed by unpaired t-tests. Assumptions of normal distributions were tested by histograms and QQ-plots. Skewed data were log-tranformed prior to t-tests. Baseline data are presented as mean ± SD or median (25; 75. percentile) for skewed data. Correlation between parameters was assessed by Pearson’s correlation coefficient and in regression analysis. A two-tailed p-value of less than 0.05 was considered statistically significant. Data were analyzed with software from Stata (Stata 11, StataCorp LP, Texas, USA).

## Results

Patient characteristics are presented in Table 1. The patients with diabetes had good glycemic control. Office systolic and diastolic BP and lipid levels were significantly lower in the diabetes group, probably due to the fact that a significantly higher proportion of the diabetes population was in antihypertensive and cholesterol lowering treatment compared with the control group. There was no difference between the two groups regarding 24-h ABPM systolic and diastolic BP, whereas 24-h pulse pressure, resting heart rate and 24-h heart rate were higher in the diabetes group. The patients with diabetes had lower creatinine levels and higher estimated glomerular filtration rate (eGFR) and urinary albumin-creatinine ratio compared with the control group. Moreover, carotid-femoral PWV was significantly higher in the diabetes group. Regarding diabetes treatment, 23 patients were treated with diet only and 67 patients were treated with one or more anti-diabetic medications. 56(84%) of these 67 patients received metformin, 12(18%) received sulfonylureas, 8(12%) insulin, 3(4%) dipeptidyl peptidase-4 inhibitors, 2 (3%) glucagon-like peptide 1.

**Table 1:** Patient characteristics

	**Diabetics**	**Controls**	**P-value**
Sex (male/female)	46/44	46/44	-
Age (years)	59 ± 10	59 ± 10	-
Diabetes duration (years) (median(IQR))	1.9(0.8;3.2)	-	-
HbA1c (%) (median(IQR))	6.5 (6.2;6.8)	5.7 (5.5;5.8)	<0.001
Body Mass Index (BMI)(kg/m^2^)	30 ± 5	26 ± 4	<0.001
Smoking (present/previous/never)	19/33/38	19/30/41	0.88
Statins (yes/no)	70/20	16/74	<0.001
Antihypertensive treatment (yes/no)	57/33	25/65	<0.001
History of cardiovascular disease (yes/no)	17/73	10/80	<0.001
Total-cholesterol (mmol/l)	4.3 ± 0.8	5.7 ± 1.0	<0.001
LDL-cholesterol (mmol/l)	2.2 ± 0.7	3.4 ± 1.0	<0.001
HDL-cholesterol (mmol/l)	1.4 ± 0.3	1.7 ± 0.6	<0.001
Triglycerides (mmol/l) (median(IQR))	1.4 (1.1;2.0)	1.2 (0.9;1.6)	<0.05
Office systolic BP (mmHg)	126 ± 12	132 ± 14	<0.01
Office diastolic BP (mmHg)	79 ± 8	84 ± 10	<0.001
Office pulse pressure (mmHg)	47 ± 10	48 ± 10	0.73
Office mean arterial pressure (mmHg)	95 ± 8.3	100 ± 11	<0.01
Office heart rate (beats/min)	66 ± 10	62 ± 10	<0.01
24-h ABPM systolic BP (mmHg)	126 ± 11	125 ± 12	0.40
24-h ABPM diastolic BP (mmHg)	75 ± 8	76 ± 8	0.26
24-h ABPM pulse pressure (mmHg)	52 ± 9	49 ± 9	<0.05
24-h ABPM mean arterial pressure (mmHg)	92 ± 8	93 ± 8	0.86
24-h heart rate (beats/min)	74 ± 10	68 ± 9	<0.01
Creatinine (μmol/liter)	72 ± 14	76 ± 14	<0.01
Estimated GFR (mL/min/1.73 m^2^) (median(IQR))	75 (66;87)	70(63;78)	<0.01
Urine albumine/creatinine ratio (mg/mmol) (median(IQR))	0.40 (0.28;0.98)	0.25 (0.17;0.40)	<0.001
Carotid-femoral pulse wave velocity (m/s)	9.3 ± 2	8.0 ± 1.6	<0.001

*IQR* Inter Quartile Range, *LDL* Low Density Lipoprotein, *HDL* High Density Lipoprotein, *BP* Blood Pressure, *ABPM* Ambulatory Blood Pressure Monitoring, *GFR* Glomerular Filtration Rate. History of cardiovascular disease: Previous acute myocardial infarction, percutaneous cardiac intervention, coronary bypass or stroke.

### Fibulin-1 and patient characteristics

Plasma fibulin-1 was associated with estimated glomerular filtration rate in the diabetes group, but not with other patient characteristics in the diabetes or control group, Table 2.

**Table 2:** **Univariate associations between plasma fibulin**-**1 concentrations and clinical variables in 90 patients with type 2 diabetes and 90 gender**- **and age**-**matched controls**

	**Type 2 diabetes patients**		**Controls**	
	**(n = 90)**		**(n = 90)**	
	**β ± SE pr μg/mL plasma fibulin-1**	**P-value**	**β ± SE pr μg/mL plasma fibulin-1**	**P-value**
Age (years)	0.5 ± 0.3	0.10	0.12 ± 0.32	0.71
HbA1c (%)	−3.8 ± 4.7	0.42	9.3 ± 9.3	0.32
Body Mass Index (kg/m^2^)	−1.1 ± 0.6	0.10	−0.01 ± 0.81	0.99
Plasma total cholesterol (mmol/l)	0.59 ± 3.84	0.88	2.33 ± 3.10	0.45
Plasma creatinine (μmol/liter)	0.24 ± 0.21	0.25	−0.41 ± 0.22	0.07
Estimated glomerular filtration rate (mL/min/1.73 m^2^)	−0.40 ± 0.19	<0.05	−0.11 ± 0.25	0.65
Ln-transformed Urine albumine/creatinine ratio (mg/mmol)	1.83 ± 3.10	0.55	4.26 ± 3.8	0.27
Office systolic BP (mmHg)	−0.06 ± 0.18	0.74	−0.14 ± 0.18	0.43
Office diastolic BP (mmHg)	−0.33 ± 0.35	0.36	−0.34 ± 0.32	0.28
Office pulse pressure (mmHg)	0.04 ± 0.23	0.86	−0.07 ± 0.26	0.80
Office heart rate (beats/min)	0.01 ± 0.20	0.98	−0.15 ± 0.34	0.66
24-h ABPM systolic BP (mmHg)	−0.31 ± 0.28	0.26	−0.13 ± 0.28	0.63
24-h ABPM diastolic BP (mmHg)	−0.78 ± 0.40	0.053	−0.24 ± 0.43	0.58
24-h ABPM pulse pressure (mmHg)	0.08 ± 0.36	0.82	−0.07 ± 0.38	0.86
24-h ABPM heart rate (beats/min)	0.22 ± 0.32	0.70	−0.40 ± 0.34	0.25

*SE* Standard Error, *BP* Blood Pressure, *ABPM* Ambulatory Blood Pressure Monitoring.

**Figure 1:**
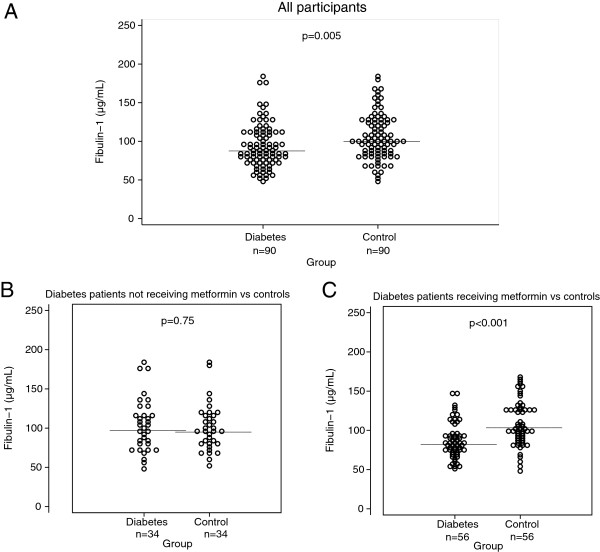
**Plasma fibulin-1 in patients with type 2 diabetes and gender- and age-matched controls.** Horizontal line indicates mean. Panel **A**: Plasma fibulin-1 in 90 patients with type 2 diabetes and 90 gender- and age-matched controls. Subgroup analyses in panel **B** and **C**: Panel **B**: Plasma fibulin-1 in the 34 diabetes patients not receiving metformin and their 34 gender- and age- matched controls. Panel **C**: Plasma fibulin-1 in the 56 diabetes patients receiving metformin and their 56 gender- and age- matched controls.

Plasma fibulin-1 levels were significantly lower in the diabetes group than in the control group, 93 ± 28 vs 106 ± 30 μg/mL, p = 0.005, as illustrated in Figure 1A. This association remained significant with adjustment for glomerular filtration rate, p < 0.05. Diabetes patients treated with metformin had significantly lower fibulin-1 levels than patients not treated with metformin, 88 ± 23 vs 103 ± 34 μg/mL, p < 0.05, despite comparable glomerular filtration rates, 78 ± 16 vs 75 ± 14 mL/min/1.73 m^2^, p = 0.28. No association was found with other anti-glycemic medications (i.e. sulfonylureas, insulin, glucagon-like-peptide-1 agonists, dipeptidyl dipeptidase-4 inhibitors) or with antihypertensive medications and lipid-lowering medications. Patients with diabetes that were not treated with metformin had plasma fibulin-1 levels comparable to the controls, 103 ± 34 vs. 100 ± 30 μg/mL, p = 0.75 (Figure 1B), whereas metformin-treated patients had markedly lower fibulin-1 levels than controls, 88 ± 23 vs. 109 ± 29 μg/mL, p < 0.001 (Figure 1C).

In the diabetes group, PWV was not significantly lower in the patients treated with metformin compared with those that were not, 9.0 ± 1.7 vs 9.6 ± 2.3 m/s, p = 0.14. Adjustment for age increased the p-value further, p = 0.58.

In the control-group, women had significantly higher plasma fibulin-1 than men, 115 ± 30 vs 97 ± 27 μg/mL, p < 0.01, whereas in the diabetes group no gender differences were seen, 96 ± 28 vs 91 ± 29 μg/mL (women vs men), p = 0.40, neither in the subgroup not receiving metformin, 101 ± 34 vs 104 ± 34 μg/mL (women vs men), p = 0.85.

### Fibulin-1 and PWV

In unadjusted analysis of the total study sample, fibulin-1 was not associated with PWV, p = 0.46. To adjust for confounding from gender in the control group and from diabetes, these parameters, with the interaction term diabetes x gender, were included in the model. With this adjustment, fibulin-1 was significantly associated with PWV, which increased 0.11 ± 0.05 m/s pr 10 μg/mL increase in plasma fibulin-1, p < 0.05. The association remained in a fully adjusted model including age, gender, mean blood pressure, heart rate, body mass index, diabetes, diabetes x gender and glomerular filtration rate. In this adjusted model, a 10 μg/mL increase in plasma fibulin was associated with 0.09 ± 0.04 m/s increase in PWV, p < 0.05. Inclusion of history of hypertension or history of cardiovascular disease did not alter the results of the analysis. The effect of metformin and the interaction term metformin × diabetes could not be assessed in this model due to collinearity between metformin and diabetes. Testing for interaction between diabetes and fibulin in this model showed borderline significance, p = 0.07. Therefore, the diabetes and control group was also evaluated separately.

**Figure 2:**
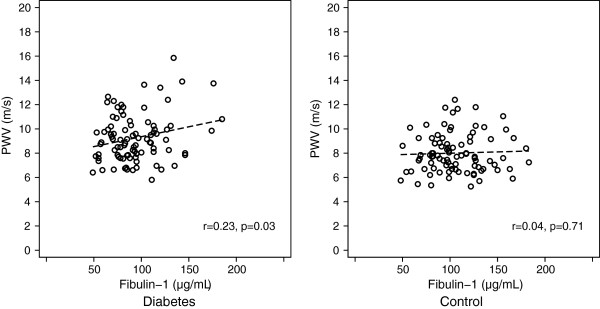
**Scatter-plots of plasma fibulin-1 and carotid femoral pulse wave velocity.** r = Pearson’s correlation coefficient.

In univariate regression analysis, plasma fibulin-1 was associated with PWV in the diabetes group, but not in the control group, Table 3 and Figure 2. The association between fibulin-1 and PWV remained significant in the diabetes group in multivariate regression analysis after adjustment for age, gender, mean arterial office BP, resting heart rate, body mass index and estimated glomerular filtration rate, Table 3. Additional adjustment for metformin did not change this association, Table 3. Inclusion of history of hypertension or history of cardiovascular disease did not alter the results of the analysis. The association between fibulin-1 and PWV did not become significant in the control group in multivariate regression analysis with adjustment for the above mentioned covariates.

**Table 3:** **Association of carotid**-**femoral pulse wave velocity and plasma fibulin**-**1 in patients with type 2 diabetes and gender and age**-**matched controls**

**Pulse wave velocity (m/s)**	**Type 2 diabetes patients (n = 90)**		**Controls (n = 90)**	
	**Coefficient ± SE pr 10** **μg/mL increase in plasma fibulin-1**	**p**	**Coefficient ± SE pr 10** **μg/mL increase in plasma fibulin-1**	**p**
Unadjusted	0.16 ± 0.07	<0.05	0.021 ± 0.057	0.70
Adjusted^a^	0.13 ± 0.06	<0.05	0.027 ± 0.041	0.52
Fully adjusted^b^	0.13 ± 0.06	<0.05	-	

*SE* Standard Error. ^a^Adjusted for age, sex, mean arterial office blood pressure, resting heart rate, body mass index and estimated glomerular filtration rate. ^b^Adjustment for the covariates in a plus metformin.

## Discussion

The first major finding of this study was a significant association between plasma fibulin-1 levels and PWV in patients with type 2 diabetes and gender- and age matched controls when adjusted for effect-modification from gender and diabetes. In stratified analysis, the association remained significant only in the diabetes group. However, as testing for interaction between diabetes and fibulin-1 was only borderline significant, no firm conclusions regarding differences in the strength of the association between PWV and fibulin-1 in the diabetes group versus the control group can be made in the present study. To the best of our knowledge, we are the first to demonstrate such an association. This suggest that, even though the association was modest, fibulin-1 might be involved in the pathophysiological processes underlying arterial stiffening.

A second major finding was that plasma fibulin-1 levels were lower in patients with type 2 diabetes compared with controls. The finding indicated, that the absolute plasma levels of fibulin-1 could not be interpreted as a simple marker of arterial stiffness, as PWV was higher in the diabetic group compared with the controls. The finding of low plasma fibulin-1 in diabetic patients was unexpected, as a previous study by Cangemi et al [11] reported markedly higher levels of plasma fibulin-1 in patients with type 2 diabetes than in non-diabetics. Plasma fibulin-1 levels should thus be interpreted in the context of potential confounding factors. Differences in duration of diabetes, gender distribution and anti-diabetic medication in the two studies may account for the observed discrepancies. The diabetes duration was not reported in the Cangemi study [11], but was probably longer in the older type 2 diabetic patients than in the present study population, and accordingly these patients may have had more severe arterial alterations than our patients. Secondly, we and others [23] found higher plasma fibulin-1 in non-diabetic females compared with males. The gender distribution (female/male) in the study by Cangemi et al [11] was 5/31 in the control group, whereas it was 44/46 in the present study. Thus, had more females been represented in the study by Cangemi et al [11], the plasma fibulin-1 level in the control population might have been higher and the difference between the diabetes and control group less pronounced. Thirdly, metformin-treated patients in our study had significantly lower fibulin-1 levels than patients not receiving metformin. The patients in the study by Cangemi et al [11] were referred for coronary bypass or carotid atherectomies, and had stopped their metformin treatment before the surgical procedure when the blood samples were drawn. The patients in the present study were in ongoing metformin treatment. As our observations are cross-sectional, causality cannot be inferred, yet if metformin had an effect on plasma fibulin-1 and this effect tapered off as the metformin was metabolized, even the short pause in relation to the surgical procedure may have raised fibulin-1 levels in the diabetes group in the study by Cangemi et al [11] to untreated values, whereas it remained lowered in our study. Clearly, this hypothesis remains speculative given our study design and requires further testing in an experimental setting. Metformin-treatment could potentially also be associated with changes in PWV or other arterial stiffness indices. A recent study found metformin treatment lowered the augmentation index in patients with non-alcohol fatty liver disease [24]. Yet, regarding PWV, intervention-studies in patients with non-alcoholic fatty liver disease [25] and with polycystic ovary syndrome [26,27] have reported conflicting results regarding metformin’s ability to lower PWV, and we did not find any association between metformin treatment and PWV.

A third finding was the lack of association of fibulin-1 with Hba1c and the traditional cardiovascular risk factors cholesterol levels and BP indices including pulse pressure. This is also in contrast with findings in the study by Cangemi et al [11] and a recent study by Scholze et al in patients with end-stage renal disease [28], where significant associations were observed between fibulin-1 and fasting plasma glucose, HbA1c, blood pressure and also plasma creatinine. It could thus be hypothesized, that the association of plasma fibulin-1 with HbA1c and BP becomes evident only after a certain disease duration with accumulated damage to the arterial wall caused by longer hyperglycemic and hypertensive exposure, whereas PWV might be a more sensitive indicator of arterial stiffening and hence could be associated with fibulin-1 levels even in the early stages of the disease. Cangemi et al also found fibulin-1 to be associated with carotid compliance, stroke volume/pulse pressure ratio, left atrial volume index and plasma NT-proBNP [11] and Scholze found fibulin-1 associated with plasma fibrinogen, plasma urea and unadjusted augmentation index [28], factors not assessed in the present study. We cannot exclude, that adjustment for these parameters might have changed the results of our analysis.

PWV was recorded under standardized examination conditions. The most important parameters to take into account when assessing PWV are age, blood pressure and heart rate [29]. Obesity may affect distance measurement. These parameters were adjusted for in the multivariate analyses.

The present findings extend previous research on the pathophysiological basis of the increased arterial stiffness found in patients with diabetes. Increased brachial-radial PWV in diabetic patients was demonstrated in 1962 [30] and interpreted as an expression of increased diabetic atherosclerosis burden. However, whereas the patchy intimal atherosclerotic lesions may contribute to arterial stiffness, atherosclerosis-independent changes in the ECM of the media layer also play a pivotal role for the increased arterial stiffness seen in diabetic patients. Increased amounts of advanced glycosylation end-products (AGEs) [5], fibronectin [6], type IV collagen [8] and hyaluronan [7] have been found in the ECM of non-atherosclerotic aortic specimens from diabetic patients compared with non-diabetics. Conversely, decreased content of elastin [9] and increased content of the elastin-cleaving metalloproteinase matrix metallopeptidase 2 [10] has been reported. These changes all contribute to increase arterial stiffness, yet the pathophysiology remains elusive. First recently, fibulin-1 has been associated with arterial alterations in diabetes patients [11]. It is known that fibulin-1 binds elastin [31], fibronectin [32] and proteoglycans [33], yet, the exact role of fibulin-1 in the arterial wall for arterial stiffness remains to be elucidated. The present findings extend on previous observations by the demonstration of an independent association between plasma fibulin-1 levels and carotid-femoral PWV, the gold standard method for assessment of arterial stiffness [3], in patients with recently diagnosed type 2 diabetes. However, due to the cross-sectional design, we cannot infer causality of the observed association between plasma fibulin-1 levels and PWV. Neither can the correspondence between arterial wall fibulin-1 and plasma fibulin-1 be assessed in this study.

Application of the mendelian randomization approach [34] in future studies might provide information regarding the existence of a causal biological association between fibulin-1 and arterial stiffness. This approach is used to make assessment of causality from observational data based on genetic variation [34,35]. A recent study reported association between a genetic marker for fibulin-2 and hypertension [36], lending support to the hypothesis that members of the fibulin family could be causally involved in arterial wall changes.

In conclusion, plasma fibulin-1 was independently associated with increased PWV in a sample of well-regulated patients with recently diagnosed type 2 diabetes and gender- and age-matched controls. Fibulin-1 might thus be involved in the pathophysiological processes underlying stiffening of the arterial wall. However, the plasma fibulin-1 level is not a simple marker of the degree of arterial stiffening, as evidenced by lower plasma fibulin-1 concentrations in the diabetes patients. Furthermore, the observed association with metformin treatment and gender confers complexity when making correlative interpretations of data employing plasma fibulin-1 levels.

These observations reflect the complex pathophysiology of arterial stiffening and underline the need for further studies to determine the exact role of fibulin-1 in arterial stiffness and cardiovascular risk.

## Competing interests

No potential conflicts of interest relevant to this article were reported.

## Authors’ contributions

EL generated the study hypothesis, developed the study design, acquired, analyzed and interpreted the data and drafted and revised the manuscript. PH developed the study design, acquired the data, critically revised the manuscript and obtained funding. JSC developed the study design, provided administrative support, obtained funding and critically revised the manuscript. STK interpreted the data and critically edited and revised the manuscript. KWH interpreted the data and critically edited and revised the manuscript. WSA interpreted the data and critically edited and revised the manuscript. TKH developed the study hypothesis and design, obtained funding, interpreted the data, provided administrative support and critically revised the manuscript. PLP developed the study hypothesis, interpreted the data, handled supervision, provided administrative support and critically revised the manuscript. LMR developed the study hypothesis, analyzed and interpreted the data, handled supervision and critically revised the manuscript. All authors read and approved the final manuscript.
